# Hypocalcemia as a Cause of Complex Febrile Seizures in a Toddler

**DOI:** 10.1155/2021/1798741

**Published:** 2021-07-13

**Authors:** Kevin Meesters, Tessa Wassenberg, Jesse Vanbesien

**Affiliations:** ^1^Department of Pediatrics, KidZ Health Castle, Vrije Universiteit Brussel, Universitair Ziekenhuis Brussel, Laarbeeklaan 101, Brussels 1090, Belgium; ^2^Pediatric Neurology Unit, KidZ Health Castle, Vrije Universiteit Brussel, Universitair Ziekenhuis Brussel, Laarbeeklaan 101, Brussels 1090, Belgium; ^3^Department of Pediatric Endocrinology, KidZ Health Castle, Vrije Universiteit Brussel, Universitair Ziekenhuis Brussel, Laarbeeklaan 101, Brussels 1090, Belgium

## Abstract

A 13-month-old boy had suffered three episodes of complex febrile seizures. At this admission, there were signs of hyperexcitability, such as Trousseau sign and QTc prolongation. A point of care blood gas analysis revealed severe hypocalcemia. Therefore, prior to administering intravenous calcium gluconate, we took blood samples to investigate the etiology of this hypocalcemia: magnesium, parathormone, and 25-hydroxyvitamin D. Since both parathormone and phosphate were significantly elevated and 25-hydroxyvitamin D was within the normal range, pseudohypoparathyroidism was diagnosed. After two years of follow-up, serum calcium had normalized in our patient under supplementation of vitamin D and calcium. He had been free of convulsions, although different febrile episodes had occurred.

## 1. Introduction

Approximately 2–5% of all Caucasian children, compared to 14% of children of Asian origin, suffer at least one febrile seizure between the ages of 6 months and 5 years. The peak incidence is at 12–18 months [1]. Following the criteria in Table 1, febrile seizures can be classified as either simple or complex [2]. Generally, laboratory or radiology investigations are not recommended after a first simple seizure [3]. Instead, the child should be screened for a febrile focus through a careful history and a thorough physical examination. In particular, signs of cerebral nervous system infection should be ruled out. Since these signs cannot be excluded reliably in young infants, a short observation period might be reasonable in children less than 18 months of age after a first simple febrile seizure [3]. Otherwise, parents should be reassured about the benign character of febrile seizures and their excellent neurological outcome [1]. On contrary, complex febrile seizures might be a presentation of an underlying disease and therefore warrant a thorough evaluation. In this article, we report the case of a toddler who had an unusual cause for his complex febrile seizures.

## 2. Case Report

A 13-month-old boy was admitted to our pediatric emergency department after four tonic-clonic seizures that had occurred during the preceding 12 hours. Each seizure lasted for around two minutes, during which time he shivered with all extremities, while he was unresponsive to both auditory and tactile stimuli. Interictally, our patient was tired, but his consciousness recovered fully. At presentation, the boy was alert, and he had a temperature of 38.6°C with mild symptoms of an upper respiratory tract infection. There were no dysmorphic features. During blood pressure measurement, tetany of his underarms was observed, which is called Trousseau sign. In the two months prior to this presentation, our patient had suffered two similar convulsions, both during febrile episodes. Therefore, an electro encephalography and a magnetic resonance scan of the brain had been performed in a regional hospital, and both were reported to be normal. Our patient took vitamin D supplements (400 international units once daily), as advised by the Belgian healthcare authorities for children up to 6 years of age, but no other medications. His further medical history was unremarkable.

**Figure 1:**
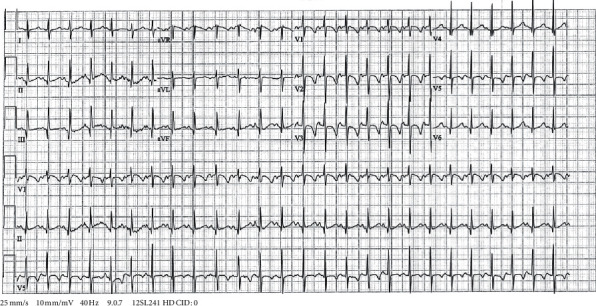
The 12-lead ECG of our patient.

A venous blood gas revealed very low ionized calcium (0.59 mmol/L). Further lab results of our patient are displayed in Table 2. His ECG (Figure 1) showed a sinus rate of 157 beats per minute with a QTc interval of 510 ms (age-specific reference: 381–447) [4]. Additional blood samples were drawn for endocrinology analyses; thereafter, intravenous calcium gluconate was administered. Subsequently, the patient was admitted to our pediatric intensive care unit, where additional doses of intravenous calcium were given. Oral vitamin D was increased to therapeutic doses, and oral calcium and magnesium supplements were commenced. No antiepileptic drugs were given. During the subsequent days, serum calcium rose slowly.

Both serum parathyroid hormone (PTH) and phosphate turned out to be significantly elevated; therefore, pseudohypoparathyroidism was diagnosed. A CT-scan of the brain was performed which showed no calcifications or other abnormalities. Our patient was referred to a geneticist and followed-up by the pediatric endocrinologist and pediatric neurologist. After two years of follow-up, serum calcium was normalized under supplementation. Different febrile episodes had occurred, but none with convulsions. Neurodevelopment was normal for his age. No known pathogenic mutations in genes usually causing pseudohypoparathyroidism were found.

## 3. Discussion

We report the case of a toddler who was admitted after his third episode of complex febrile seizures. Since electrolyte disturbances can cause convulsions, the analysis of electrolytes should be considered after complex febrile seizures, particularly in children with risk factors for these disturbances such as vomiting and diarrhea [5]. In our patient, we found Trousseau sign, which is a specific sign for hypocalcemia. Owing the important role in signal transduction, a low level of free calcium provokes convulsions, paresthesias, and tremors [6].

98-99% of the body calcium content is stored in the bones; the rest resides in the intracellular and extracellular spaces [7]. Of serum calcium, approximately 50% is ionized and thereby available for physiological processes and renal filtration. The other half is inactivated as this is bound to molecules, mainly albumin and to a far lesser extent to citrate and phosphate. Therefore, deviations in these binding molecules affect the free calcium concentration. Furthermore, in acidosis, protein bound calcium dissociates resulting in a raised ionized calcium, opposed to alkalosis in which free calcium binds to proteins [8].

Under physiologic circumstances, PTH is released after a decrease in either ionized calcium or magnesium, both detected by the calcium sensing receptor (CaSR) that is located in the parathyroid gland and renal tubules. PTH enhances calcium reabsorption through reciprocal phosphate excretion in the kidney. Furthermore, PTH stimulates osteoclasts to release calcium from the skeleton. Last, PTH stimulates 1-alpha hydroxylase that converts 25-hydroxyvitamin D into its active substrate 1,25-dihydroxyvitamin, which increases serum calcium through intestinal absorption and renal tubular reabsorption.

The first step in evaluating a patient for hypocalcemia should be confirming low ionized calcium through direct analysis. If this is unavailable, free calcium can be estimated using formulas correcting for serum albumin. After pseudohypocalcemia, which is a low serum but normal ionized calcium, has been ruled out, additional blood samples should be collected for the analyses as given in Table 3, ideally prior to treatment initiation. Generally, a disorder in calcium metabolism can be identified using a limited number of analyses [7, 9].

In addition to marked hypocalcemia, both serum phosphate and PTH were significantly elevated in our patient. This is the classic triad of pseudohypoparathyroidism, provided that serum 25-hydroxyvitamin D and magnesium are both within the normal range [10]. Pseudohypoparathyroidism is a syndrome characterized by end-organ resistance to PTH, caused by either sporadic or hereditary mutations in exons of the GNAS gene. The prevalence of pseudohypoparathyroidism is unknown but was estimated 1 : 20000 in the USA, and 1 : 100000 in Denmark. Most patients have normal PTH and calcium levels at birth, and patients are often unrecognized in the absence of obvious clinical features. These may include skeletal defects, such as long bone shortening, spinal stenosis, brachydactyly, and short stature as an adult. Furthermore, subcutaneous ossifications and obesity are common. Apart from the resistance to PTH, affected patients may have resistance to other hormones, such as TSH, GHRH, gonadotrophins, calcitonin, and glucagon. The main therapeutic goal in these patients is to correct ionized calcium by oral vitamin D, calcium, and often magnesium supplements. However, given its rareness in children and the involvement of different hormone axes, patients with pseudohypoparathyroidism should be referred to a pediatric endocrinologist for further management.

## Figures and Tables

**Table 1:** Classification of febrile seizures [2].

Simple febrile seizure	Complex febrile seizure
All of the following:	One of the following:
(i) Generalized	(i) Focal or prolonged generalized
(ii) Less than 15 minutes	(ii) Longer than 15 minutes
(iii) No recurrence in a 24-hour period	(iii) Recurrent within 24 hours
—	(iv) Associated postictal abnormalities
In a child aged 6 months to 5 years with no neurologic deficits, who has fever at least in the immediate postacute period.	—

**Table 2:** Laboratory parameters of our patient.

Parameter	On admission	1^st^ day	2^nd^ day	Reference range
Hemoglobin	11.0	—	—	9.8–13.8 g/dL
Hematocrit	32.3			29.4–42.0%
MCV	82.4			68–90 fL
Platelets	406 000			158–470000/mm^3^
White blood cells	17 000			3500–17000/mm^3^
Absolute neutrophil count	11 798			1700–8700/mm^3^
Lymphocytes	3281			2700–8700/mm^3^

Serum Na	139	136	142	135–145 mmol/L
Serum K	4.8	4.3	4.4	3.5–5.0 mmol/L
Serum Ca	1.36	1.39	1.63	2.17–2.44 mmol/L
Serum phosphate	3.34	2.73	2.79	1.26–2.10 mmol/L
Serum Mg	0.68	0.59	0.63	0.65–1.05 mmol/L

Urea	16	6	—	11–36 mg/dL
Creatinine	0.26	0.22		<0.70 mg/dL
Albumin	45	40		36–52 g/L
CRP	21.4	55.4		<5 mg/L
Alkalic phosphatase	219	230		145–320 U/L
Gamma glutamyl transferase	12	—		6–19 U/L
Amylase	55			50–130 U/L
Parathormone	442.9			15–65 ng/L
Thyroid stimulating hormone	5.08			0.27–4.2 mIU/L
Free thyroxine	14.8			11.0–24.0 pmol/L
25-Hydroxyvitamin D	51.9			20–50 microg/L

pH	7.369	7.345	7.386	7.35–7.45
pCO_2_	35.7	38.4	34.1	36–44 mmHg
pO_2_	52.4	34.6	67.1	90–100 mmHg
Bicarbonate	20.6	21.0	20.4	22–26 mmol/L
Base excess	−4.1	−4.4	−4.0	−2–+2
Hb saturation	83.7	57.6	92.4	—
Methemoglobin	0.7	0.3	0.1	0.5–3.0%
Na	138	135	140	135–145 mmol/L
K	5.5	4.3	4.2	3.5–5.0 mmol/L
Cl	103	103	107	96–109 mmol/L
Ionized Ca	0.59	0.63	0.73	1.15–1.30 mmol/L

**Table 3:** Investigations in a child with hypocalcemia [7, 9].

Analysis	Interpretation
Ionized calcium	Physiologically active and available for glomerular filtration and excretion.
Synonym: free calcium	If unavailable, estimate free calcium by formulas correcting for albumin.

Serum parathormone (PTH)	Reflects activity of the calcium sensing receptor.
	Low levels suggest hypoparathyroidism or hypomagnesemia.
	High levels reflect either impaired calcium absorption or end-organ resistance to PTH (pseudohypoparathyroidism).

Serum phosphate	Inversely correlated with PTH, therefore an indirect measure of PTH activity.

Serum magnesium	Calcium and magnesium homeostasis are strongly correlated by the calcium sensing receptor.
	If low, correct and reassess ionized calcium thereafter.

25-OH-Vitamin D	Best marker of vitamin D storage in the body.

Alkaline phosphate, gamma GT	Markers of bone turnover. Often elevated by rising PTH, as this stimulates osteoclasts to release calcium from the skeleton.

## Data Availability

The data used to support the findings of this study are included within the article.
